# Time-reversed ultrasonically encoded optical focusing through highly scattering *ex vivo* human cataractous lenses

**DOI:** 10.1117/1.JBO.23.1.010501

**Published:** 2018-01-10

**Authors:** Yan Liu, Yuecheng Shen, Haowen Ruan, Frank L. Brodie, Terence T. W. Wong, Changhuei Yang, Lihong V. Wang

**Affiliations:** a California Institute of Technology, Department of Electrical Engineering, Pasadena, California, United States; b California Institute of Technology, Andrew and Peggy Cherng Department of Medical Engineering, Pasadena, California, United States; c University of California, San Francisco, Department of Ophthalmology, San Francisco, California, United States

**Keywords:** focusing light through turbid media, wavefront shaping, adaptive optics, optical phase conjugation, time-reversed ultrasonically encoded optical focusing, cataract, amblyopia

## Abstract

Normal development of the visual system in infants relies on clear images being projected onto the retina, which can be disrupted by lens opacity caused by congenital cataract. This disruption, if uncorrected in early life, results in amblyopia (permanently decreased vision even after removal of the cataract). Doctors are able to prevent amblyopia by removing the cataract during the first several weeks of life, but this surgery risks a host of complications, which can be equally visually disabling. Here, we investigated the feasibility of focusing light noninvasively through highly scattering cataractous lenses to stimulate the retina, thereby preventing amblyopia. This approach would allow the cataractous lens removal surgery to be delayed and hence greatly reduce the risk of complications from early surgery. Employing a wavefront shaping technique named time-reversed ultrasonically encoded optical focusing in reflection mode, we focused 532-nm light through a highly scattering *ex vivo* adult human cataractous lens. This work demonstrates a potential clinical application of wavefront shaping techniques.

Normal development of the visual pathways in the central nervous system relies on clear images being projected on the retina throughout the first year of life. Disruption of this can lead to the development of amblyopia—a condition in which individuals, despite having structurally normal eyes, have intractable poor vision due to the underdevelopment of the cortical visual system.^1^^–^^3^

A cataract is a clouding of the normally transparent crystalline lens in the eye, and it scatters light coming toward a retina. Cataract causes half of blindness and 33% of visual impairment worldwide. Congenital cataract occurs approximately one in every 2500 live births.^4^ Since no clear images are projected to the retinas of the infants with such a disease, early diagnosis and treatment of congenital cataract is critical for the prevention of amblyopia.^5^^,^^6^

Currently, the standard of care is to perform cataract removal surgery within the first month of life,^5^^,^^6^ to minimize the effects of cataract on the normal development of the visual pathways. The infant is usually left aphakic, i.e., without a physiological lens in the eye, and it relies on a contact lens. Unfortunately, a common complication of cataract extraction is the development of glaucoma (termed aphakic glaucoma, which involves damaging of the optic nerve that leads to vision loss). While the precise mechanism for this complication is not well understood, it has been shown that earlier surgery leads to an increased risk.^7^^–^^9^

Ultimately, current management of congenital cataract puts the doctor in a difficult position: the cataract needs to be removed promptly to prevent amblyopia, but the surgeon knows that aphakic glaucoma could lead to equally profound vision loss after the cataractous lens is removed. Although the risk of aphakic glaucoma can be reduced eightfold by delaying the surgery until four months of life, evidence shows that this delay would lead to more severe amblyopia.^5^^–^^7^

A potential solution to this problem would be having the ability to focus light through the opaque cataractous lens to stimulate the retina, thereby preventing amblyopia and giving the eye more time to mature (particularly the eye’s drainage system, since glaucoma usually happens when fluid builds up in the anterior section of an eye, which increases the intraocular pressure and damages the optic nerve). This approach would allow the cataractous lens removal surgery to be delayed and thereby greatly reduce the risk of aphakic glaucoma.^7^

To focus light through opaque cataractous lenses for retina stimulation, we use wavefront shaping, which includes a class of methods that employs scattered photons for focusing light through highly scattering media, such as biological tissue.^10^^–^^13^ These methods work by shaping the wavefront of an incident light field, so that the scattered light can constructively interfere at locations of interest to form optical foci.^14^ Three types of wavefront shaping techniques have been developed, including feedback-based wavefront shaping,^14^^,^^15^ transmission matrix measurement,^16^^,^^17^ and optical phase conjugation (OPC)/time reversal.^18^^–^^22^ Among them, OPC achieves the highest focusing speed for a given number of wavefront sensing and control elements (runtime <10  ms for $>10^{5}$ elements^23^^–^^25^), by determining the required wavefront globally instead of stepwise.^26^ This feature makes OPC most promising for *in vivo* applications, where speckles decorrelate quickly due to physiological motions.^23^^,^^27^

OPC focuses light inside scattering media by first measuring and then phase conjugating (time reversing) the scattered light field emitted from a guide star,^11^^,^^28^^–^^30^ which is positioned at a targeted focusing location deep inside a scattering medium. Here, we use focused ultrasound to noninvasively provide a (virtual) guide star,^31^^–^^33^ which is freely addressable within tissue. Due to the acousto-optic effect, a portion of the light passing through the ultrasonic focus changes its frequency by an amount equal to the ultrasonic frequency. These so-called ultrasound-tagged photons emitted from the virtual guide star (ultrasonic focus) are then scattered as they propagate through the turbid medium toward our camera. By measuring the wavefront of the ultrasound-tagged light and then performing OPC, we generate a phase-conjugate version of the ultrasound-tagged light, which partially retraces its original trajectory back through the scattering medium and converges to the ultrasonic focus (the source of the ultrasound-tagged light), as if time has been reversed. This focusing technique based on ultrasound-guided OPC is known as time-reversed ultrasonically encoded (TRUE) optical focusing.^31^^–^^33^

The cataractous lens used in our experiment was harvested from a 68-year-old male donor at the University of California San Francisco Medical Center. A photograph of the cataractous lens is shown in Fig. 1(a). Because of the strong scattering of light in the lens, we cannot observe the “CALTECH” characters underneath. To quantify the extinction coefficient $\mu_{t}$ of the cataractous lens, we measured the transmission of collimated ballistic light through a tissue slice, which was attached to a glass slide [Fig. 1(b)]. Rather than using the whole lens, a thin slice (thickness L=100  μm, cut with a vibratome) was used to reduce the number of scattered photons.^34^ To reject the scattered light, the distance between the sample and a photodetector was kept long (2.6 m), and an iris with a diameter of 1.5 mm was used. According to Beer’s law, the transmitted light power received by the photodetector $P_{1}=P_{0}t_{1}t_{2}\;\exp(-\mu_{t}L)t_{3}$, where $P_{0}$ is the incident light power on the glass slide and $t_{1}$, $t_{2}$, and $t_{3}$ are the transmission coefficients of the air-glass, glass-tissue, and tissue-air interfaces, respectively [Fig. 1(b)]. To reduce the unknown variables by normalization, we also measured the transmitted light power $P_{2}$ through another tissue slice with a thickness of 2L. Since $P_{2}=P_{0}t_{1}t_{2}\;\exp(-\mu_{t}2L)t_{3}$, we obtain $\mu_{t}=\ln(P_{1}/P_{2})/L=32\pm 4\;{\mathrm{mm}}^{-1}$. The thickness of the cataractous lens that we focused light through was 3.5 mm [Fig. 1(a)], which equaled 112 mean free paths ($1{/\mu }_{t}$).

**Fig. 1:**
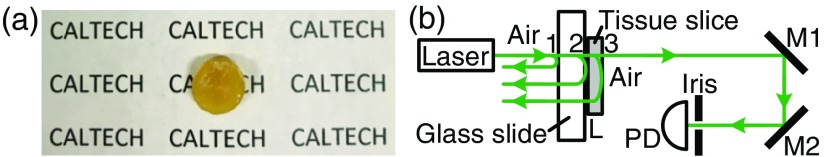
Illustration of the turbidity of an *ex vivo* human cataractous lens. (a) The lens was so scattering that the “CALTECH” characters underneath cannot be observed. (b) Schematic of the setup to measure the extinction coefficient of the cataractous lens. M, mirror; PD, photodetector.

To focus light through the cataractous lens, we employed a custom-built reflection-mode TRUE focusing system^35^ (schematically shown in Fig. 2). We first measured the phase map of the ultrasound-tagged light field using heterodyne holography^22^^,^^33^^,^^36^ and then phase conjugated this field by displaying the conjugate phase map on a spatial light modulator (SLM) that modulated the phase of light. Following time reversal, the phase conjugated light would converge to the ultrasonic focus, thus forming an optical focus. To stimulate a retina in practice, we will park the ultrasonic focus either on or sufficiently close to the retina. In this proof-of-concept experiment, we parked the ultrasonic focus close to the retina (Fig. 2), so we could then remove the scattering retina to directly image the optical focus by an imaging system (composed of objective OBJ1, tube lens TL, and camera CAM2, see Fig. 2 inset) and verify that TRUE focusing worked. In actual applications, we can validate the TRUE focus by observing an increase in the measured ultrasound-modulated light signal, compared with the case without doing wavefront shaping. The distance between the lens and the ultrasonic focus was 15 mm, which is the typical distance between the lens and the retina of an infant.

**Fig. 2:**
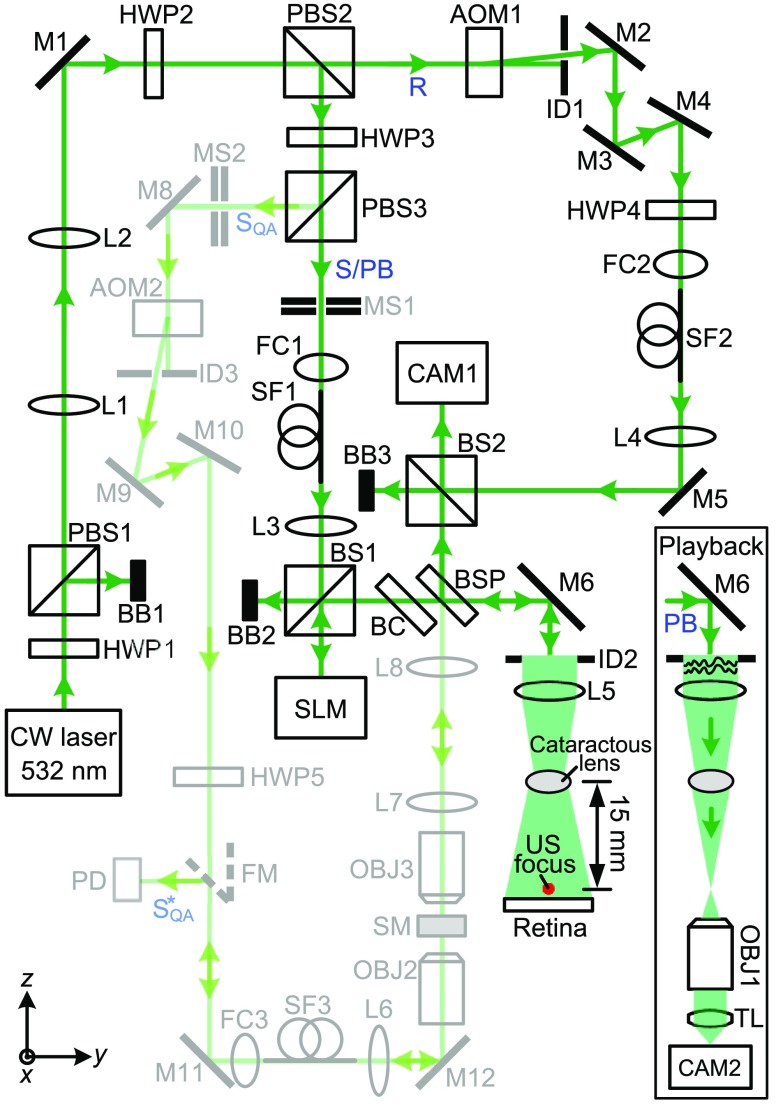
Schematic of the setup for focusing light through an *ex vivo* human cataractous lens. The optical path in light green was used for assessing and ensuring the performance of the OPC setup on a daily basis. The inset shows the schematic of the setup for observing the TRUE focus. AOM, acousto-optic modulator; BB, beam block; BC, beam compensator; BS, cube beamsplitter; BSP, plate beamsplitter; CAM, camera; CW, continuous-wave; FC, fiber coupler; FM, flip mirror; HWP, half-wave plate; ID, iris diaphragm; L, lens; M, mirror; MS, mechanical shutter; OBJ, objective; PB, playback beam; PBS, polarizing beamsplitter; PD, photodiode; R, reference beam; S, sample beam; SF, polarization-maintaining single-mode optical fiber; $S_{\mathrm{QA}}$, sample beam for quality assurance of the OPC system; $S_{\mathrm{QA}}^{*}$, conjugate of $S_{\mathrm{QA}}$. SLM, spatial light modulator; SM, scattering medium (two layers of tapes); TL, tube lens; US, ultrasound.

In Fig. 2, the output of a 200 mW, 532-nm continuous-wave laser (Excelsior-532-200, Spectra-Physics) was split into a sample beam (S)/playback beam (PB) and a reference beam (R). Both beams were spatially filtered by single-mode fibers and collimated. The frequency of R was up-shifted by 50  MHz+10  Hz by acousto-optic modulator AOM1 before R was reflected to scientific CMOS camera CAM1 (pco-edge 5.5, PCO, 15-ms exposure time) by beamsplitter BS2. In the other arm, S/PB beam reflected from the SLM (Pluto, Holoeye) and mirror M6 illuminated the cataractous lens, with an intensity of $15\;{\mathrm{mW}/\mathrm{cm}}^{2}$. A portion of the light back-scattered from a cow retina was tagged by a 50 MHz focused ultrasonic field, collected by lens L5 (ACL50832U, Thorlabs), and then reflected to camera CAM1 by plate beam splitter BSP (50T/50R). On CAM1, the ultrasound-tagged light interfered with reference beam R, with a beat frequency of 10 Hz. By triggering the camera at four times the beat frequency (40 Hz) and recording successive interferograms ($I_{0}$, $I_{\pi /2}$, $I_{\pi }$, $I_{3\pi /2}$), we were able to reconstruct the phase map of the ultrasound-tagged light by $\varphi =\mathrm{Arg}[(I_{0}-I_{\pi })+i(I_{\pi /2}-I_{3\pi /2})]$, where Arg[z] computes the principal value of the argument of complex number z. A 30-time averaging for each phase of the interferogram was used to improve the signal-to-noise ratio (SNR). To achieve OPC, we displayed the conjugate phase map of φ on the SLM, which was positioned at the mirrored position of the camera sensor relative to beamsplitter BSP. The wavefront-shaped light would then converge to the ultrasonic focus after passing through the cataractous lens (Fig. 2 inset). We used an iterative TRUE focusing scheme^35^^,^^37^^,^^38^ to increase the SNR and resolution by repeating the TRUE focusing procedure using a previously established TRUE focus. Eight iterations were employed, and the SNR was doubled.

A portion of the phase map displayed on the SLM to achieve TRUE focusing is shown in Fig. 3(a). Only the central 200×200  pixels out of 1920×1080  pixels are shown due to space constraint. The histogram of the whole phase map shows that the phase values are nearly uniformly distributed between −π and π [Fig. 3(b)], following the statistics of a fully developed speckle. Figures 3(a) and 3(b) show that the wavefront observed here is much more complex than that in traditional adaptive optics.^39^ This capability to tackle complex wavefront associated with highly scattering media is enabled by a reliable guide star and the large pixel counts ($>10^{6}\text{ pixels}$) of both the wavefront sensor (scientific CMOS camera) and the wavefront modulator (SLM) used by our technique.

**Fig. 3:**
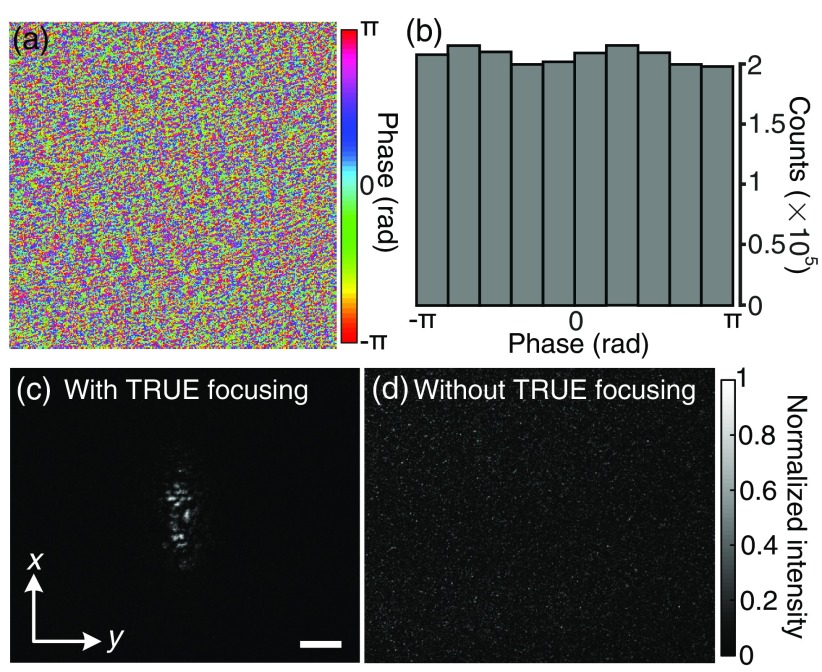
Focusing light through an *ex vivo* human cataractous lens. (a) A portion of the phase map displayed on the SLM to achieve TRUE focusing. (b) Histogram of the phase map. (c) Image of the TRUE focus observed on camera CAM2. (d) No focus was observed when we shifted the phase map displayed on the SLM horizontally by 10 pixels to break the time-reversal symmetry. Scale bar, 100  μm.

When the phase map partially shown in Fig. 3(a) was displayed on the SLM, the wavefront-shaped light was focused through the cataractous lens; the optical focus observed on camera CAM2 outside the water tank is shown in Fig. 3(c). The full width at half maximum focal spot size is 52  μm along the y-direction and 173  μm along the x-direction, which is the acoustic axis direction. The spot size may be reduced by using more iterations of TRUE focusing, and the spot size along the acoustic axis direction can be reduced by using a pulsed laser and a shorter ultrasonic pulse. The average intensity inside the focus is 13 times higher than the average intensity of the surrounding background. This focusing contrast is ∼9% of the theoretical value, and the discrepancy may be due to SLM curvature, imperfect reference beams, and imperfect alignment between the SLM and camera CAM1. In a control experiment, we shifted the phase map displayed on the SLM horizontally by 10 pixels to break the time-reversal symmetry, and no focus was observed [Fig. 3(d)], as expected.

Focusing light inside scattering media using wavefront shaping is an area of active research because it breaks the optical diffusion limit^40^^,^^41^ and promises to revolutionize biophotonics by enabling noninvasive deep-tissue optical imaging,^42^ manipulation,^43^ and therapy. Recently, we succeeded in focusing 532-nm light through 25-mm-thick *ex vivo* chicken tissue, as well as through 96-mm-thick tissue-mimicking phantoms,^21^ demonstrating the great potential of OPC-based wavefront shaping for biomedicine. For *in vivo* applications, the system runtime should be shorter than the speckle correlation time associated with living tissue, which is on the order of 1 ms due to blood flow.^23^^,^^27^^,^^44^ Although high-speed systems are being actively developed,^23^^–^^25^^,^^36^^,^^45^^–^^47^ unless we reduce the number of controls, the speeds need to be further improved for *in vivo* deep-tissue applications. In contrast, since there are no blood vessels in human lens or in retina layers at the fovea and the cataractous lens can be static for a relatively long time, the speckle correlation time would be much longer. Therefore, focusing light through human cataractous lens *in vivo* can be a promising application for wavefront shaping techniques.
